# Supplementary material for: Highly Stable Docetaxel-Loaded Nanoparticles Based on Poly(D,L-lactide)-b-Poly(ethylene glycol) for Cancer Treatment: Preparation, Characterization, and In Vitro Cytotoxicity Studies
Source: Polymers (Basel). 2023 May 13;15(10):2296. doi: 10.3390/polym15102296 (PMC10221316; doi:10.3390/polym15102296)
Supplement: Supplementary file 1 [file polymers-15-02296-s001.zip › polymers-2343815-supplementary.pdf]

Supplementary Materials

Highly Stable Docetaxel-Loaded Nanoparticles Based on Poly(D,L-lactide)-*b*-Poly(ethylene glycol) for Cancer Treatment: Preparation, Characterization, and In Vitro Cytotoxicity Studies

Ekaterina V. Kuznetsova [1,*], Nikita G. Sedush [1,2], Yulia A. Puchkova [1], Sergei V. Aleshin [1], Evgeny V. Yastremsky [3], Alexey A. Nazarov [4] and Sergei N. Chvalun [1,2]

[1] National Research Center "Kurchatov Institute", Moscow 123182, Russia; nsedush@gmail.com (N.G.S.); yellow_jk@mail.ru (Y.A.P.); neos.1991@gmail.com (S.V.A.); s-chvalun@yandex.ru (S.N.C.)
[2] Enikolopov Institute of Synthetic Polymeric Materials, Russian Academy of Sciences, Moscow 117393, Russia
[3] Shubnikov Institute of Crystallography, Federal Science Research Center "Crystallography and Photonics", Russian Academy of Science, Moscow 119333, Russia
[4] Department of Chemistry, Lomonosov Moscow State University, Moscow 119991, Russia
* Correspondence: kuznetsova.kate992@gmail.com

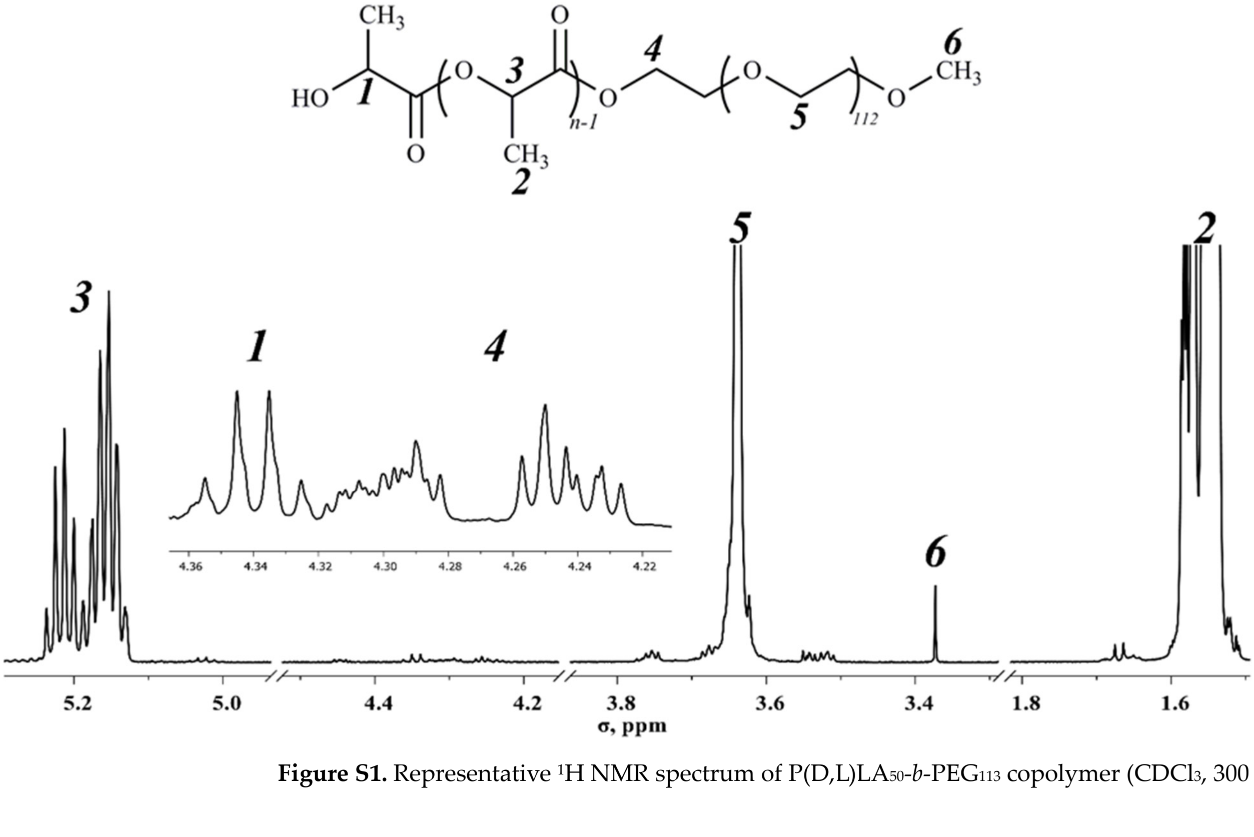

Figure S1. Representative ^{1}H NMR spectrum of P(D,L)LA_{50}-*b*-PEG_{113} copolymer ($CDCl_3$, 300 MHz).

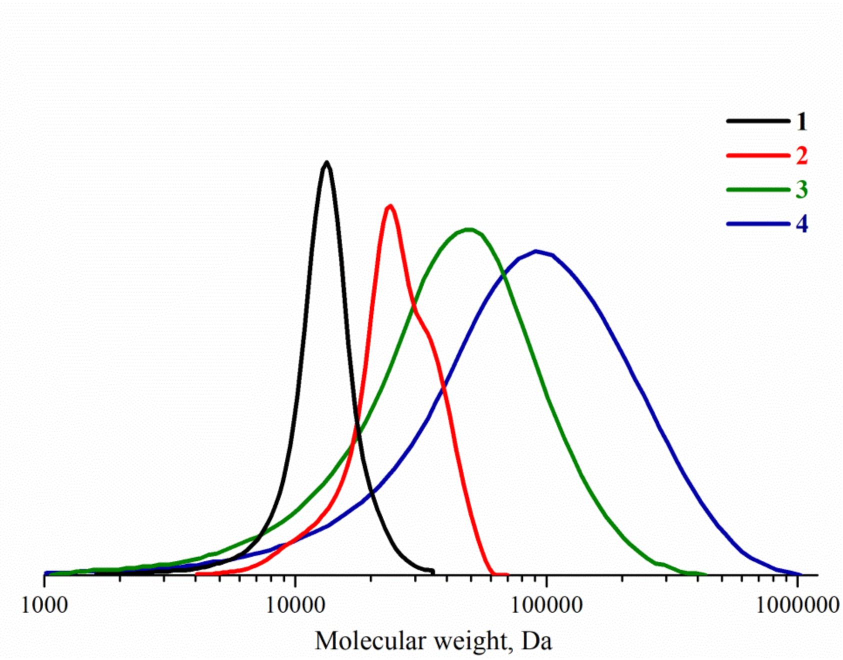

Figure S2. Chromatograms of the synthesized block copolymers: 1 – P(D,L)LA$_{50}$-*b*-PEG$_{113}$, 2 – P(D,L)LA$_{180}$-*b*-PEG$_{113}$, 3 – P(D,L)LA$_{680}$-*b*-PEG$_{113}$, 4 – P(D,L)LA$_{1230}$-*b*-PEG$_{113}$.

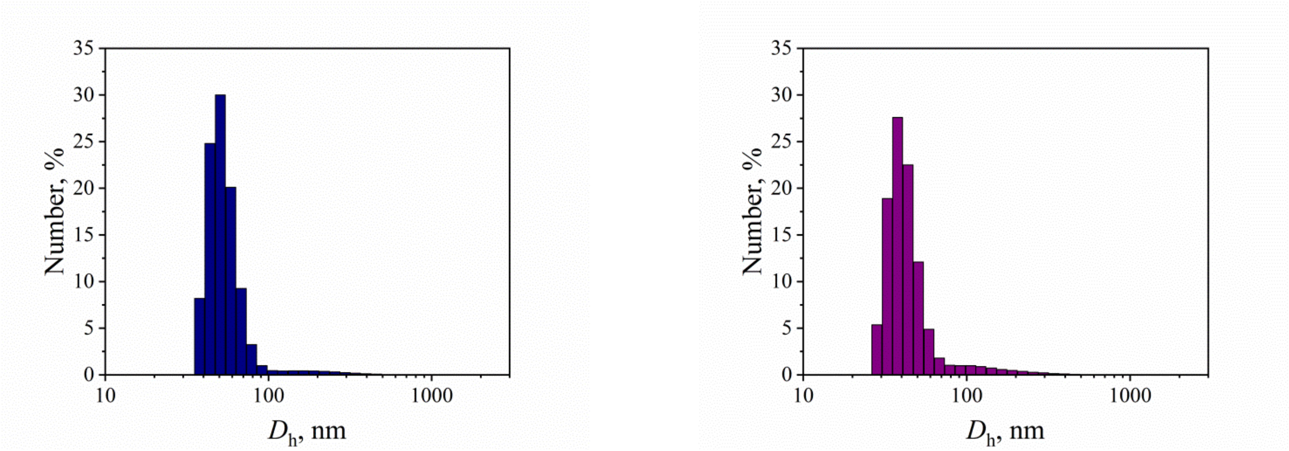

a **b**

Figure S3. DLS number size distribution curves for diluted aqueous suspensions with concentration of 0.1 g/L based on (**a**) P(D,L)LA$_{50}$-*b*-PEG$_{113}$ and (**b**) P(D,L)LA$_{180}$-*b*-PEG$_{113}$ copolymers.

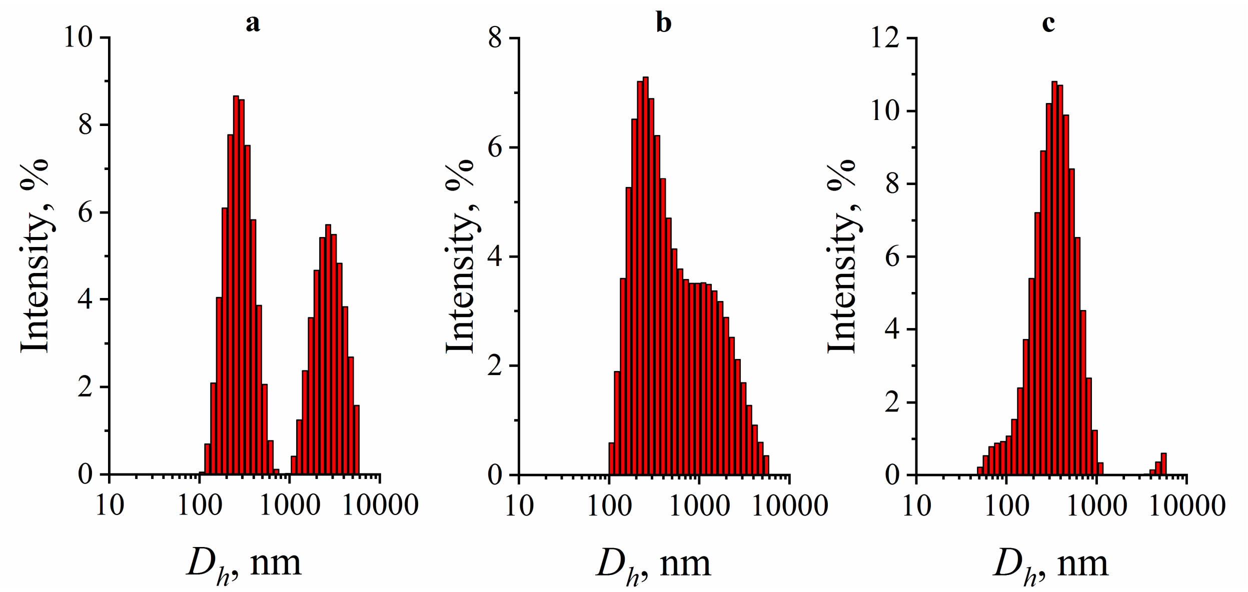

Figure S4. DLS intensity size distribution curves for aqueous suspensions of docetaxel-loaded P(D,L)LA_{1230}-*b*-PEG_{113} nanoparticles after freeze-drying with D(-)-mannitol and following reconstitution. The ratio of lyoprotectant:NPs was (**a**) 1:4, (**b**) 1:1 and (**c**) 2.5:1.

Evaluation of parameters of P(D,L)LA_n-*b*-PEG_{113} nanoparticles. The values of core–corona interface area (s_{int}) of the P(D,L)LA_n-*b*-PEG_{113} nanoparticles (NPs) were calculated from the diameter of poly(D,L-lactide) P(D,L)LA core (*D*) evaluated from TEM according to the following equation:

$$s_{int} = \frac{3}{\rho \cdot (D/2)},$$

where ρ = 1.25 g/cm^3 is the bulk density of PLA.

The values of tethering density of hydrophilic PEG chains (σ) on the P(D,L)LA core surface of the P(D,L)LA_n-*b*-PEG_{113} NPs were calculated according to the equation:

$$\sigma = \frac{1}{s_{int}} = \frac{N_{agg}}{4\pi(D/2)^2},$$

where s_{int} is the P(D,L)LA core area corresponding to one PEG chain, N_{agg} is the aggregation number of NP.

The values of N_{agg} of the P(D,L)LA_n-*b*-PEG_{113} NPs were calculated according to the following equation:

$$N_{agg} = \frac{(D/2)^3 4\pi N_A \rho}{3M_0 N_{P(D,L)LA} 10^{21}},$$

where N_A is the Avogadro constant, M_0 is the molecular weight of lactide monomer unit, $N_{P(D,L)LA}$ is the polymerization degree of the P(D,L)LA block.
